# When Did *Carcharocles megalodon* Become Extinct? A New Analysis of the Fossil Record

**DOI:** 10.1371/journal.pone.0111086

**Published:** 2014-10-22

**Authors:** Catalina Pimiento, Christopher F. Clements

**Affiliations:** 1 Florida Museum of Natural History, Gainesville, Florida, United States of America; Department of Biology, University of Florida, Gainesville, Florida, United States of America; Smithsonian Tropical Research Institute, Balboa, Panama; 2 Institute of Evolutionary Biology and Environmental Studies, The University of Zurich, Zurich, Switzerland; New York Institute of Technology College of Osteopathic Medicine, United States of America

## Abstract

*Carcharocles megalodon* (“Megalodon”) is the largest shark that ever lived. Based on its distribution, dental morphology, and associated fauna, it has been suggested that this species was a cosmopolitan apex predator that fed on marine mammals from the middle Miocene to the Pliocene (15.9–2.6 Ma). Prevailing theory suggests that the extinction of apex predators affects ecosystem dynamics. Accordingly, knowing the time of extinction of *C. megalodon* is a fundamental step towards understanding the effects of such an event in ancient communities. However, the time of extinction of this important species has never been quantitatively assessed. Here, we synthesize the most recent records of *C. megalodon* from the literature and scientific collections and infer the date of its extinction by making a novel use of the Optimal Linear Estimation (OLE) model. Our results suggest that *C. megalodon* went extinct around 2.6 Ma. Furthermore, when contrasting our results with known ecological and macroevolutionary trends in marine mammals, it became evident that the modern composition and function of modern gigantic filter-feeding whales was established after the extinction of *C. megalodon*. Consequently, the study of the time of extinction of *C. megalodon* provides the basis to improve our understanding of the responses of marine species to the removal of apex predators, presenting a deep-time perspective for the conservation of modern ecosystems.

## Introduction

*Carcharocles megalodon* (“Megalodon”) was the largest shark that ever lived [1]. Based on its dentition, distribution and associated fauna, it has been suggested that this species could reach up to 18 m of total length, was a cosmopolitan apex predator and fed on cetaceans [1]–[7]. Its gigantic size and abundant fossil record has made this shark a charismatic example of extinct marine megafauna. However, despite its popularity and widespread fossil record, remarkably little is known about its extinction.

It has been widely stated in the literature that the extinction of apex predators can trigger cascading effects through entire food webs and impact ecosystem composition and function [8]–[9]. Concurrently, it has been demonstrated that the elimination of large sharks produces broad marine ecosystem degradation [10]. In modern marine systems, apex predators, especially large sharks, are significantly declining throughout the global oceans [11]–[12]. The study of the extinction of apex predatory sharks is therefore of significant interest.

A fundamental step towards understanding the effects of an extinction event is to know when it occurred. However, identifying the exact time of extinction of a species is notoriously difficult because the fossil record is inherently incomplete. Hence, the absence of records of a species does not necessarily mean that it is extinct, but could instead reflect preservation bias, spatially heterogeneous populations, sampling effort, or as yet uncovered fossil remains [13]–[15]. Such issues imply that the last recorded occurrence of a species, or “Last Appearance Date” (LAD) as a proxy for time of extinction provides an inherently biased estimate [16].

In order to identify the time of extinction of a species, multiple methods based on the temporal distribution of the most recent sighting events (or analogously the fossil record), have been proposed (see [14] for a review). Many of these remain poorly tested; however, the Optimal Linear Estimation (OLE) model [13] has been shown to provide accurate estimates of when a species can be considered to have become extinct [15].

Given that *C. megalodon* has an abundant fossil record, cosmopolitan distribution and high trophic level, its extinction is an ideal case study to better understand the ecological and macroevolutionary responses of marine species to top-down control release. It is generally reported that fossils of this species range from the middle Miocene (15.9–11.6 Ma) to the Pliocene (5.3–2.6 Ma), with some unconfirmed reports (i.e. considered to be unreliable, see Supplementary Information) from the Pleistocene (2.6–0.01 Ma) [1]–[7]. However, the time of extinction of *C. megalodon* has never been quantitatively assessed before.

Here, we synthesize the most recent records of *C. megalodon* from the literature and scientific collections, and probabilistically infer the time by which it became extinct by using the OLE model. Because OLE has only been previously used to tackle the problem of inferring the time of extinction of modern species [13], in this study we extend its use to the analogous issue of inferring extinction events in deep time (for a more detailed explanation of the reasons why we used this method, see the Materials and Methods section). Based on our estimated time of extinction and known ecological and macroevolutionary trends in cetaceans, we further outline the possible effects of this event, providing the basis for better understanding of the responses of marine species to the removal of apex predators.

## Results

Based on the known age range of *Carcharocles megalodon*, we considered any post-Miocene occurrence as part of the most recent records of the species, and treated them as historic sightings to be used in the OLE calculation. Accordingly, we identified a total of 53 most recent records, which are made available in the Paleobiology Database (PaleoBioDB, http://paleobiodb.org). However, we only included in our analysis a subset of data consisting of the 42 records deemed to be reliable (Table S1).

The age of the fossils is not absolute, but instead falls within a range, with an upper and lower time estimate. To account for this unbiased uncertainty, we re-sampled the fossil data 10,000 times, bootstrapping the timing of each record from a uniform distribution between its upper and lower age. Hence, the results presented will to some extent be sensitive to the uniform distribution used in this analysis. Nevertheless, given that the actual age of the fossils is equally likely to have occurred anywhere within their age span, the use of this distribution is justified.

Because OLE infers the time by which a species can be considered extinct from the temporal distribution of the most recent sighting events [13], we regarded the time of the extinction event to most likely have occurred between the oldest inferred date of extinction (the first point in time at which the species can be considered as extinct), and the modal value (the most frequently inferred time of extinction). We use the modal value, as opposed to the mean, as it gives a more accurate reflection of the skewed distribution of inferred dates of extinction.

**Figure 1:**
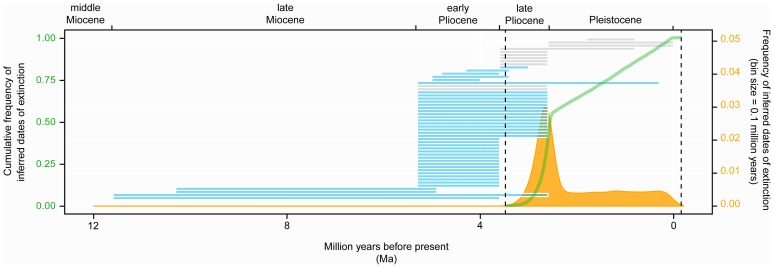
Temporal distribution of the inferred dates of extinction of *Carcharocles megalodon* using the Optimal Linear Estimation (OLE) model bootstrapped 10,000 times. The orange area shows the distribution of inferred dates of extinction through time, whereas the green line shows the cumulative frequency of inferred dates of extinction. The modal peak represents the point in time by which the species was most likely to have gone extinct (2.6 Ma). Approximately 50% of simulations fell before the modal peak of inferred dates of extinction (2.6 Ma), whereas the remaining 50% are roughly evenly distributed between the mode and the present day. The two vertical dashed lines indicate the most recent and oldest inferred dates of extinction (160,000 years in the future and 3.5 Ma respectively). The horizontal bars represent the time range of each fossil occurrence. The blue bars are the occurrences used in OLE. The grey bar represents the occurrences that failed the age evaluation process and were not used in the analysis.

Results from applying OLE to our set of most recent records show that the modal inferred time of extinction is 2.6 Ma. This suggests that *C. megalodon* was unlikely to have survived beyond this time, with extinction time thus likely to have occurred between 3.5 Ma (oldest inferred extinction date), and this modal value (Figure 1). Approximately 50% of the simulations inferred the extinction event to have occurred before 2.6 Ma (modal value), with the remaining 50% of simulations being roughly uniformly distributed between 2.6 Ma and 0.1 Ma (Figure 1). In a very small proportion of simulations (1.5%), the inferred date of extinction fell after 0.1 Ma. In six simulations (0.06%) the inferred date of extinction fell after the present day (and thus the species could not be considered as extinct). However, because in the vast majority of the 10,000 simulations (>99.9%) the extinction time was inferred to have occurred before the present day, we reject the null hypothesis (that the species is extant) and the popular claims of present day survival of *C. megalodon*.

## Discussion

Our analysis suggests that the extinction of *Carcharocles megalodon* most likely occurred around Pliocene–Pleistocene boundary (∼2.6 Ma, modal value). Interestingly, subsequent to this time and throughout the Pleistocene, baleen whales (Cetacea, Mysticeti) reached modern gigantic sizes [17]–[19]. This mysticete composition contrasts with assemblages that were contemporaneous with *C. megalodon*, and that included mostly small-bodied species [17]. Because body size correlates with ecological functions, it has been further proposed that the faunal turnover observed in cetaceans during the Pliocene-Pleistocene boundary resulted in additional niches occupied by baleen whales [17], [20]–[21].

Fossils of mysticetes are frequently found along with *C. megalodon* teeth. This has led to the hypothesis that they interacted in ancient marine communities [e.g. 5]. Whether or not *C. megalodon* preyed upon mysticetes needs further investigation. However, based on the inferred time of extinction of *C. megalodon* and the known ecological and macroevolutionary trends of cetaceans, we propose that the modern composition and function of gigantic filter-feeding whales established after the extinction of *C. megalodon*.

The controlling factors within ecosystems are not limited to top-down processes. Bottom-up effects are also important drivers in marine ecosystem dynamics [e.g. 22] and should be taken into consideration when studying the ecological and macroevolutionary trends of marine organisms. Of relevance to this work, diatom diversity and temperature changes (indicated by oxygen stable isotopes) have been associated with the changes in diversity of marine mammals throughout the Cenozoic [23]. However, it is not clear if these bottom-up processes drove the evolution of modern gigantic sizes in filter-feeding whales. Future work contrasting top-down and bottom-up processes with mysticetes body size trends are needed to discern the drivers of the evolution of gigantism in cetaceans.

Even though ecosystems are not driven entirely by only one type of control, the study of the extinction of apex predators has great potential to advance the understanding of the responses of marine species to top-down control release. Despite the limitations and uncertainties of the fossil record, the study of the time of extinction of *C. megalodon* provides a baseline to understand the establishment of the modern structure and function of gigantic filter-feeding whales. Furthermore, the methods used here could be applied to quantitatively assess the time of extinction of other fossil organisms, ultimately helping elucidate the causes and effects of extinction events.

## Materials and Methods

### Data collection

Based on the known fossil record of *Carcharocles megalodon*, we considered any post-Miocene occurrence as part of the most recent records of the species. Accordingly, we gathered all known post-Miocene records from the literature and scientific collections. First, we searched for *C. megalodon* occurrences in the PaleoBioDB using the following parameter: Species name  =  *Carcharocles megalodon*. The PaleoBioDB includes all known synonyms (e.g. *Carcharodon megalodon*) in the search. There, each data record represents a fossil collection. A collection is any set of fossils whose exact geographic and stratigraphic position and date of collection cannot be distinguished, regardless of when they were described, and by whom. With relevance to this study, a data record or collection in the PaleoBioDB is treated as an occurrence (i.e. data record  =  collection  =  occurrence) and each of them are linked to one or more supporting references. Second, we searched for additional *C. megalodon* reports in ISI Web of Science (http://webofknowledge.com) GeoRef (http://geoscienceworld.org), Shark-References (http://shark-references.com) and Google Scholar (http://scholar.google.com) using the search terms: Megalodon OR *Carcharodon* AND *megalodon* OR *Carcharocles* AND *megalodon*.

### Dataset construction

With the data collected, we created a comprehensive and updated dataset of the most recent records of *C. megalodon*. These include 15 pre-existing PaleoBioDB records and 38 new records, for a total of 53 post-Miocene records. They accessible in the PaleoBioDB, and can be found when doing a “Full Search” of “Fossil collection records” using the species name *Carcharocles megalodon* under “Taxon Name”, and restricting the search to Pliocene to Holocene under “Time interval”. These records are part of the ongoing PaleoBioDB Data Archive # 20 (http://goo.gl/PpIh0G) and are also presented in the Supporting Information.

### Data filtering

To determine which of these records to include in our analyses, we performed a standardized evaluation as follows: for each reference reporting a post-Miocene occurrence of *C. megalodon*, we assessed if the age of the record was clearly stated in the text. In addition, we studied a number of supplementary references that further documented or refined the age of the localities from which some of the specimens were recovered. Finally, we visited the most relevant museum collections housing the specimens referenced in the literature. There we analyzed their morphology to assess for signs of re-deposition, examined their labels, and explored the collection databases to verify the age assignments. Whenever a reference did not meet the requirements of our evaluation process, we did not include that record in our analysis (Table S2). Additional information, including all records and their evaluations can be found in the Supporting Information section (Text S1). Furthermore, more detailed information and supporting references can be accessed when clicking on the PaleoBioDB# of each record.

### Analysis

As a result of our age evaluation process (Text S1), we selected the records deemed to be reliable (Table S1) and treated them as historic sightings to apply the OLE model. We employed this method, rather than other methods that have previously been used to infer extinction from the fossil record [24]–[27] because of two main reasons: (1) OLE has been thoroughly tested and shown to produce accurate estimates of the date of extinction of a species, and to outperform many similar methods [14]–[15], [28]. (2) The problem of inferring the time of extinction of a species from the fossil record is mathematically analogous to inferring the extinction of modern species from sighting record [e.g. 14], [29]–[31].

OLE infers the time by which a species can be considered as extinct from the temporal distribution of the most recent sighting events [13]. This method assumes that, regardless of the distribution of the complete sighting record, the most recent *k* sighting events have the form of a Weibull extreme value distribution, and infers the shape parameter, and thus the timing of extinction, from the joint distribution of these *k* sightings [13]. In effect, the method uses the spacing between recorded sighting events to infer the time at which, probabilistically, no more sightings will occur (the species can be considered extinct).

From Roberts and Solow [13], the Optimal Linear Estimation takes *k* sighting events and estimates 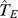, the time at which the extinction can be inferred, using the form:
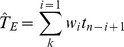
Where the weight vector *w*, length *k*, is given by:



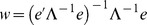


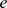
 being a vector of *k* 1′s and 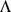 is a symmetric *k* by *k* matrix with typical element:



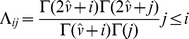
where 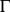 is the gamma function and 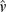 is an estimate of the shape parameter of the Weibull extreme value distribution given by



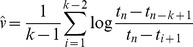


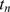
 being the 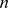 times a species is observed over the period of time 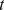. So under the assumption a species is extinct, the upper bound of an approximate 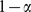 confidence interval for 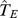 is



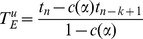
Where



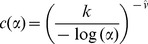



Note that in Solow [32] there is an error in the final equation, but presented above is the correct version.

There has been some debate as to what should be considered the *k* most recent sighting events [31]–[32], however recent work has shown that increasing the number of sighting events used in the calculation provides increasing accuracy of inferred dates of extinction [31]. Because of this, we use the most recent sighting events as presented in the Supporting information.

As in Clements et al. [15], we disregarded the confidence intervals produced by the technique as they have been shown to be very wide (Figure S1) and hence, concentrated on the distribution of the inferred extinction dates. Therefore, we regarded the modal value of the 10,000 estimates as the time by which *C. megalodon* is most likely to have gone extinct, with the exact date to have occurred between the mode and the oldest estimate. All simulations were made using the R statistical software [33] and the code is made available in the Supporting Information section (Files S1–S2).

## Supporting Information

Figure S1 **Distribution of inferred extinction date (red line), as well as upper (blue) and lower (green) 95% confidence intervals through time.** The modal peaks of the upper and lower 95% confidence intervals fall close to the modal peak of the inferred date of extinction; however, the tail of the upper 95% confidence interval extends far beyond the present day, with the latest estimate falling 2.6 million years in future.
(TIF)

File S1 **Code to calculate the distribution of inferred dates of extinction of** ***Carcharocles megalodon*** **using the Optimal Linear Estimation (OLE) model.**
(R) Click here for additional data file.

File S2 **Data set of the Post-Miocene occurrences of** ***Carcharocles megalodon*** **used in the analysis.**
(CSV)

Table S1 **Post-Miocene records of** ***Carcharocles megalodon*** **included in the Optimal Linear Estimation (OLE) model (click on the PaleoBioDB# for more details).**
(PDF)

Table S2 **Post-Miocene records of** ***Carcharocles megalodon*** **excluded from Optimal Linear Estimation (OLE) model (click on the PaleoBioDB# for more details).**
(PDF)

Text S1 **Age evaluation of** ***Carcharocles megalodon*** **post-Miocene records (click on the PaleoBioDB# for more details).**
(PDF)
