# Correction: When Did *Carcharocles megalodon* Become Extinct? A New Analysis of the Fossil Record

**DOI:** 10.1371/journal.pone.0117877

**Published:** 2015-01-30

**Authors:** The PLOS ONE Staff

In the Materials and Methods section, there are errors in the first and third equations of the section titled “Analysis.” The complete, correct first equation is:

$${\hat{T}}_{E}=\sum_{i=1}^{k}w_{i}t_{n-i+1}$$

The complete, correct third equation is:

$$\Lambda_{ij}=\frac{\Gamma (2\hat{v}+i)\Gamma (\hat{v}+j)}{\Gamma (\hat{v}+i)\Gamma (j)}j\leq i$$
