# Supplementary figures and images for: When Did Carcharocles megalodon Become Extinct? A New Analysis of the Fossil Record
Source: PLoS One. 2014 Oct 22;9(10):e111086. doi: 10.1371/journal.pone.0111086 (PMC4206505; doi:10.1371/journal.pone.0111086)

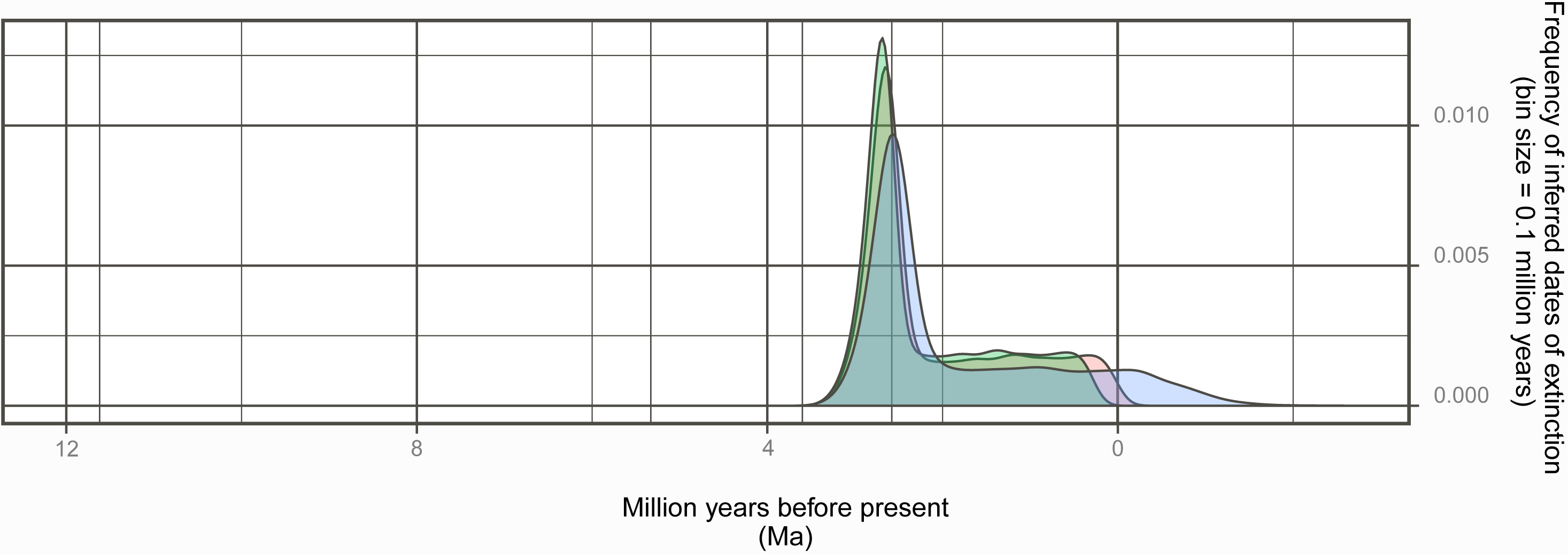

Supplement: Figure S1 — Distribution of inferred extinction date (red line), as well as upper (blue) and lower (green) 95% confidence intervals through time. The modal peaks of the upper and lower 95% confidence intervals fall close to the modal peak of the inferred date of extinction; however, the tail of the upper 95% confidence interval extends far beyond the present day, with the latest estimate falling 2.6 million years in future. (TIF) [file pone.0111086.s001.tif]
