# Supplementary material for: When Did Carcharocles megalodon Become Extinct? A New Analysis of the Fossil Record
Source: PLoS One. 2014 Oct 22;9(10):e111086. doi: 10.1371/journal.pone.0111086 (PMC4206505; doi:10.1371/journal.pone.0111086)
Supplement: Table S1 — Post-Miocene records of Carcharocles megalodon included in the Optimal Linear Estimation (OLE) model (click on the PaleoBioDB# for more details). (PDF) [file pone.0111086.s004.pdf]

Table S1. Post-Miocene records of *Carcharocles megalodon* included in the OLE (click on the PaleoBioDB# for more details)

PaleoBio DB #	Country	Formation	Locality	Max age	Min age
18548	USA (FL)	Bone Valley	Kingsford Min	10.3	4.9
18577	USA (FL)	Bone Valley	Payne Creek Mine	5.3	3.6
28039	New Zealand	Tangahoe	Hawera	3.6	3.0
45478	USA (FL)	Bone Valley	Four Corners Mine	5.3	3.6
50068	USA (CA)	San Mateo	Lawrence Canyon	10.3	4.9
50343	Australia	Loxton Sand	Sunlands Pumping	4.3	3.4
51328	Australia	Whaler's Bluff	Dutton Way	5.3	3.6
51335	Australia	Grange Burn	Fossil Rock Stack	5.0	4.0
51414	Australia	Cameron Inlet	Cameron Inlet	5.3	2.6
52582	USA (NC)	Yorktown	Lee Creek Mine	5.3	3.6
97234	USA (CA)	Towsley	Elsmere Canyon	5.3	3.6
107489	Venezuela	Paraguana	El Yacural	5.3	3.6
136597	Libya	Main Vertebrate Spot	Main Vertebrate Spot	5.3	3.6
151641	USA (FL)	Bone Valley	Fort Green Mine SW	5.3	3.6
151679	USA (FL)	Bone Valley	North Palmetto Mine	5.3	3.6
151680	USA (FL)	Bone Valley	Achan Mine	5.3	3.6
151681	USA (FL)	Bone Valley	Palmetto Mine (Agrico)	5.3	3.6
151682	USA (FL)	Bone Valley	Chicora Mine	5.3	3.6
151704	Ecuador	Onzole/Borbon	Punta la Gorda	5.3	2.6
151705	Ecuador	Onzole/Borbon	Punta la Colorada	5.3	2.6
151880	Antigua & Barbuda	Highlands	Darby Sink	5.3	2.6
151881	Cuba	Canímar	Loma Fines	11.6	2.6
151888	Spain	Arenas	Bonares-Casa del Pino	5.3	3.6
152190	New Zealand	Pipiriki	Pipiriki	4.8	3.6
152191	New Zealand	Wanganui	Wanganui	5.3	0.3
152233	Spain	Can Picafort	Can Picafort	5.3	2.6
152237	Italy	Castell'Arquato	Castell'Arquato	5.3	2.6
152241	Italy	Miano	Miano	5.3	2.6
152242	Italy	Colli Piacentini	Colli Piacentini	5.3	2.6

152243	Italy	Maiatico	Maiatico	5.3	2.6
152244	Italy	Tra Lorenzana e Lari	Tra Lorenzana e Lari	5.3	3.6
152246	Italy	Pienza	Pienza	5.3	2.6
152247	Italy	Siena	Siena	5.3	2.6
152249	Italy	Colline Pisane	Colline Pisane	5.3	2.6
152381	USA (FL)	Tamiami	East Coast Aggregates	5.3	3.6
152545	USA (CA)	Capistrano	San Juan Capistrano	11.6	3.6
154111	Angola	Luanda	Farol das Lagostas	5.3	2.6
154112	Australia	Basal Black Rock	Beaumaris	5.0	3.4
154113	Portugal	Touril Complex	Cré outcrop	5.3	3.6
154114	Chile	Bahia Inglesa	Bahia Inglesa	16.0	3.6
154117	Mexico	Tirabuzon/Gloria	Corkscrew Hill	5.3	2.6
154118	Mexico	La Salada	Rancho Algodones	5.3	2.6
