# Supplementary material for: When Did Carcharocles megalodon Become Extinct? A New Analysis of the Fossil Record
Source: PLoS One. 2014 Oct 22;9(10):e111086. doi: 10.1371/journal.pone.0111086 (PMC4206505; doi:10.1371/journal.pone.0111086)
Supplement: Table S2 — Post-Miocene records of Carcharocles megalodon excluded from Optimal Linear Estimation (OLE) model (click on the PaleoBioDB# for more details). (PDF) [file pone.0111086.s005.pdf]

Table S2. Post-Miocene records of *Carcharocles megalodon* excluded from OLE (click on the PaleoBioDB# for more details)

PaleoBio DB#	Country	Formation	Locality	Max age	Min age
55451	Madagascar	Madagascar Basin	Ocean bottom	1.8	0.8
74791	South Africa	Hondeklip Bay	Hondeklip Bay	5.3	2.6
152398	Japan	Daito	Daito Limestone	3.6	0.8
152404	French Polynesia		Station 281	2.6	0.0
152481	United Kingdom	Red Crag	Essex (Dovercourt)	3.6	2.6
152482	United Kingdom	Red Crag	Suffolk (Felixstowe)	3.6	2.6
152483	United Kingdom	Red Crag	Suffolk (Woodbridge)	3.6	2.6
152484	United Kingdom	Red Crag	Suffolk (Sutton)	3.6	2.6
154115	Mexico	San Diego	La Joya Exit	3.6	2.6
155229	USA (CA)	Repetto or Pico	Torrance	2.6	0.0
152485	USA (CA)	Purisima	Bolinas Bay	5.3	2.6
