# Supplementary material for: When Did Carcharocles megalodon Become Extinct? A New Analysis of the Fossil Record
Source: PLoS One. 2014 Oct 22;9(10):e111086. doi: 10.1371/journal.pone.0111086 (PMC4206505; doi:10.1371/journal.pone.0111086)
Supplement: Text S1 — Age evaluation of Carcharocles megalodon post-Miocene records (click on the PaleoBioDB# for more details). (PDF) [file pone.0111086.s006.pdf]

Records included in the OLE (Table S1):

PaleoBioDB# 18548: Early Pliocene age clearly explained in text. However, when studying specimens in the collections of the Florida Museum of Natural History (FLMNH), a Hemphillian age was regarded for this locality. Locality information gathered after contrasting specimen's numbers [UF 17872, UF 217140] from reference with FLMNH database.

PaleoBioDB# 18577: Early Pliocene age clearly explained in text and confirmed when studying specimens in the collections of the FLMNH. Locality information gathered after contrasting specimens' number [UF 24715] from reference with FLMNH database.

PaleoBioDB# 28039: Waipipian age (New Zealand Stage) clearly explained in text. Date confirmed in the National Paleontological Collection Database of New Zealand (NPC, http://data.gns.cri.nz/npc/).

PaleoBioDB# 45478: Early Pliocene age clearly explained in text and confirmed when studying specimens in the collections of the FLMNH. Locality information gathered after contrasting specimens' number [UF 217225] from reference with FLMNH database.

PaleoBioDB# 50068: Hemphillian age clearly explained in text.

PBD# 50343: Kalimnan age (Australian Stage) clearly explained in text. Specimens [P15216, 2 specimens] further studied from the collections of the British Museum of Natural History (NHM-London) confirmed this occurrence and its age.

PaleoBioDB# 51328: Early Pliocene age clearly explained in text.

PaleoBioDB# 51335: Age 4 to 5 Ma (based on Sr/Sr) clearly explained in text.

PaleoBioDB# 51414: Pliocene age clearly explained in text.

PaleoBioDB# 52582: Early Pliocene age clearly explained in text. Material further studied in the collections of the U. S. National Museum of Natural History (USMNH) [USNM 175642, USNM 256332-256335, USNM 279258-279262, USNM 279267, USNM 279279-279283, USNM 279407, USNM 279415, USNM 279424-279425, USNM 279432, USNM 281392, USNM 287699, USNM 287708-287709, USNM 292978, USNM 292990, USNM 293746, USNM 293748, USNM 293752, USNM 293754, USNM 293758, USNM 295339, USNM 299740, USNM 339918, USNM 348165-348166, USNM 348168, USNM 348178, USNM 348265, USNM 348277, USNM 348278-348280, USNM 348283, USNM 348286, USNM 348305, USNM 348307, USNM 348308, USNM 348337, USNM 348375, USNM 348381, USNM 348406, USNM 348410, USNM 348418, USNM 348420, USNM 348429, USNM 348431, USNM 348436, USNM 348445, USNM 348446, USNM 348448-348457, USNM 348460-348462, USNM 348466, USNM 348471-348472, USNM 348512, USNM 348514-348517, USNM 348521, USNM 348524, USNM 348525, USNM 348530, USNM 348532, USNM 348535, USNM 348540, USNM 350928, USNM 350929, USNM 350932, USNM 350935, USNM 350936, USNM 350939, USNM 350940, USNM 350941- 350942, USNM 350991-351100, USNM 353735, USNM 353738, USNM 355714, USNM 355715-355716, USNM 355718, USNM 355726, USNM 355728, USNM 355736 ,USNM 355740, USNM 355742-355743, USNM 355746, USNM 355749, USNM 355752, USNM 355756, USNM 355760, USNM 355761, USNM 355763, USNM 355767,

USNM 355772, USNM 355776-355777, USNM 355780-355782, USNM 355822, USNM 355854- 355855, USNM 355858, USNM 355860, USNM 355864, USNM 355866, USNM 355870, USNM 355871, USNM 355872, USNM 355874, USNM 355880, USNM 355885-355887, USNM 355890, USNM 355894-355895, USNM 355904, USNM 355917, USNM 355928, USNM 355938, USNM 356968-356969, USNM 356972-356973, USNM 356975-356976, USNM 356980, USNM 392159-392162, USNM 392171, USNM 482310, USNM 482311, USNM 482313, USNM 530191-530192, USNM 530606, USNM 530607, USNM 530608, USNM 531643, USNM 537789, USNM182108] confirms this occurrence and its age.

PaleoBioDB# 97234, 107489: Early Pliocene age clearly explained in text.

PaleoBioDB# 136597: Early Pliocene age clearly explained in text.

PaleoBioDB# 151641, 151679-151682: Early Pliocene age clearly explained in text and confirmed when studying specimens in the collections of the FLMNH. Locality information gathered after contrasting specimens' numbers [UF 234583, UF 228479-228480, UF 17850, UF 17839, UF 229804, UF 229807, UF 17840] from reference with FLMNH database.

PaleoBioDB# 151704-151705: Considered Miocene in reference, however, most recent works have considered this locality to be Pliocene in age, clearly explaining it in the text. Specimens [P59279-59282] studied from the collections of the NHM-London confirmed this occurrence and its age.

PaleoBioDB# 151880: Pliocene age clearly explained in text.

PaleoBioDB# 151881: Late Miocene to Pliocene age for this site, clearly explained in recent work from same author.

PaleoBioDB# 151888: Early Pliocene age clearly explained in text.

PaleoBioDB# 152190: Opoitian age (New Zealand Stage) clearly explained in text. Date confirmed in the NPC Database.

PaleoBioDB# 152191: Opoitian to Castlecliffian age (New Zealand Stages) clearly explained in text. Date confirmed in the NPC Database. Material [P27667] further studied in the collections of the NHM-London confirms this occurrence and its age.

PaleoBioDB# 152233, 152237, 152241-152243: Pliocene age clearly explained in text.

PaleoBioDB# 152244: Early Pliocene age clearly explained in text.

PaleoBioDB# 152246-152247, 152249: Pliocene age clearly explained in text.

PaleoBioDB# 152381: Early Pliocene clearly explained in text. Specimens [UF 259902-259902, UF 259907-259908] studied from the collections of the FLMNH confirmed this occurrence and its age.

PaleoBioDB# 152545: Late Miocene to early Pliocene age based on supporting references from the same locality. One specimen [LACM 129982] from Natural History Museum of Los Angeles County (LACM) studied.

PaleoBioDB# 154111: Pliocene age clearly explained in text. Additional specimens [P8950, P48412] further studied from the collections of the NHM-London confirm this occurrence and its age.

PaleoBioDB# 154112: Cheltenhamian to Kalimnan age (Australian Stages) clearly explained in text. Additional specimens [P13932, P5209] further studied from the collections of the NHM-London confirm this occurrence and its age.

PaleoBioDB# 154113: Zanclean age clearly explained in text.

PaleoBioDB# 154114: According with reference, locality dated between 4.5 and 2.6 Ma. However, most recent studies place the Bahia Inglesa Formation between the middle Miocene and the early Pliocene. Additional material [69 uncatalogued specimens] studied in the collections of the Museo Nacional de Historia Natural de Chile (MNHN-Chile), confirm this occurrence.

PaleoBioDB# 154117: Early to middle Pliocene according with text. Pliocene age in other records of the Tirabuzon Formation in the PaleoBioDB. Further material [LACM 29065, LACM 29076] studied from the collections in the LACM confirms this occurrence, and a Pliocene age.

PaleoBioDB# 154118: Pliocene age clearly explained in text.

Records excluded from the OLE (Table S2):

PaleoBioDB# 55451: Unconfirmed Pleistocene age. Specimens [CP 79010, CP 79012] collected from the center of manganese nodules and exhibited in the Muséum national d'Histoire naturelle (MNHN-France). Original label states age needs to be tested.

PaleoBioDB# 74791: Late Pliocene age in text, however, correction made to early or middle Pliocene age by subsequent publication. Text also states it should take into consideration that sediments in which the fossils are found could be much older and reworked.

PaleoBioDB# 152398: Late Pliocene to early Pleistocene age stated in text. Other records of this formation in the PaleoBioDB are early Pleistocene. Cannot confirm this occurrence due to poor quality images and lack of additional information available.

PaleoBioDB# 152404: Pleistocene age stated in text based on tickles and rate growth of the manganese dioxide layer embedded in teeth. Because only part of the crown of the teeth is preserved, assignment to Megalodon dubious.

PaleoBioDB# 152481-152484: Age of the Red Crag Formation has been widely assigned to the late Pliocene, however, the specimens [P39586, P44103-44104, P49459, P47381-47382, P6487, P46760, P41199, P37737-9, P27519, P39004-5, P1197, P34373-4, P46030, P1197, P34710-1, P34643, P24866, P39006] studied from the NHM-London, are notably eroded and most likely reworked from older sediments.

PaleoBioDB# 154115: Upper Pliocene age stated in the text, which also coincides with other reports of the San Diego Formation in the PaleoBioDB. Two teeth [LACM 156334, SDNHM 29742] from this formation studied in the collections of the LACM and the San Diego Natural History Museum (SDNHM). Both look eroded, suggesting redeposition.

PaleoBioDB# 155229: No information on the formation. Paper mentions Torrance Locality from Los Angeles basin but unable to identify geologic unit based on the information provided.

PaleoBioDB# 152485: Specimen from the Purisima Formation in Bolinas Bay. The Purisima Formation ranges from the Miocene to the Pliocene. However, it has not been possible to locate this unit in Bolinas Bay and hence, provenance of this record not reliable. Studied specimens [UCMP 219502] from this unit in University of California Museum of Paleontology (UCMP) regarded to Miocene age.

Other, no post-Miocene records (not included in dataset):

PaleoBioDB# 155341: Lower Pliocene age stated in text, but late Miocene age stated in Date NPC Database.

PaleoBioDB# 155343: Pliocene age stated in text, but late Miocene age stated in Date NPC Database.
